# Supplementary material for: Conservation of Nuclear Receptor DAF-12 with Evidence for Ligand Diversification in Plant-Parasitic Nematodes
Source: bioRxiv. 2026 Sep 7:2026.09.03.748874. Preprint. [Version 1] doi: 10.64898/2026.09.03.748874 (PMC13622478; doi:10.64898/2026.09.03.748874)

bioRxiv preprint doi: https://doi.org/10.64898/2026.09.03.748874; this version posted September 7, 2026. The copyright holder for this preprint (which was not certified by peer review) is the author/funder, who has granted bioRxiv a license to display the preprint in perpetuity. It is made available under a CC-BY 4.0 International license.

FIGURE S1

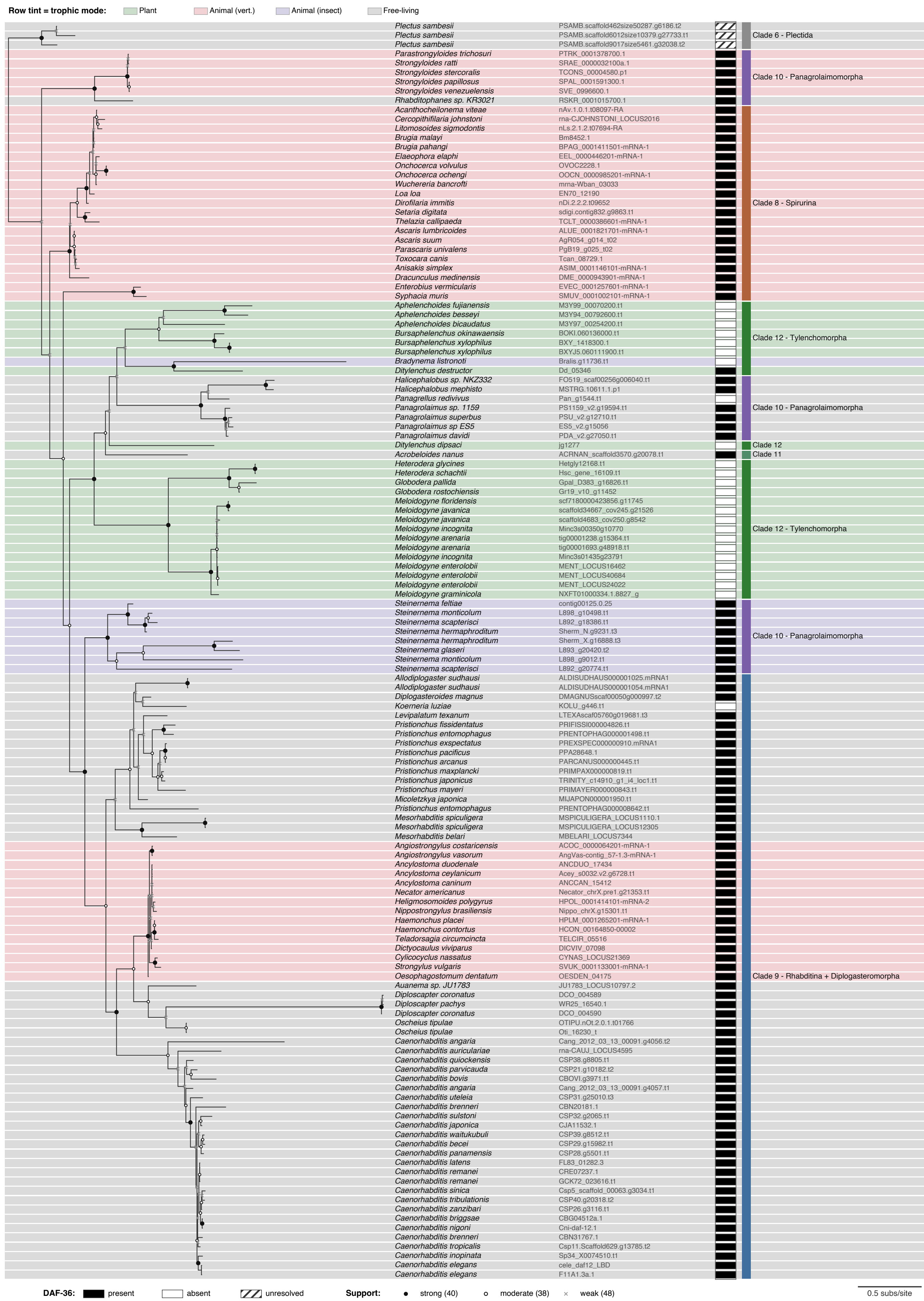

bioRxiv preprint doi: https://doi.org/10.64898/2026.09.03.748874; this version posted September 7, 2026. The copyright holder for this preprint (which was not certified by peer review) is the author/funder, who has granted bioRxiv a license to display the preprint in perpetuity. It is made available under a CC-BY 4.0 International license.

FIGURE S2

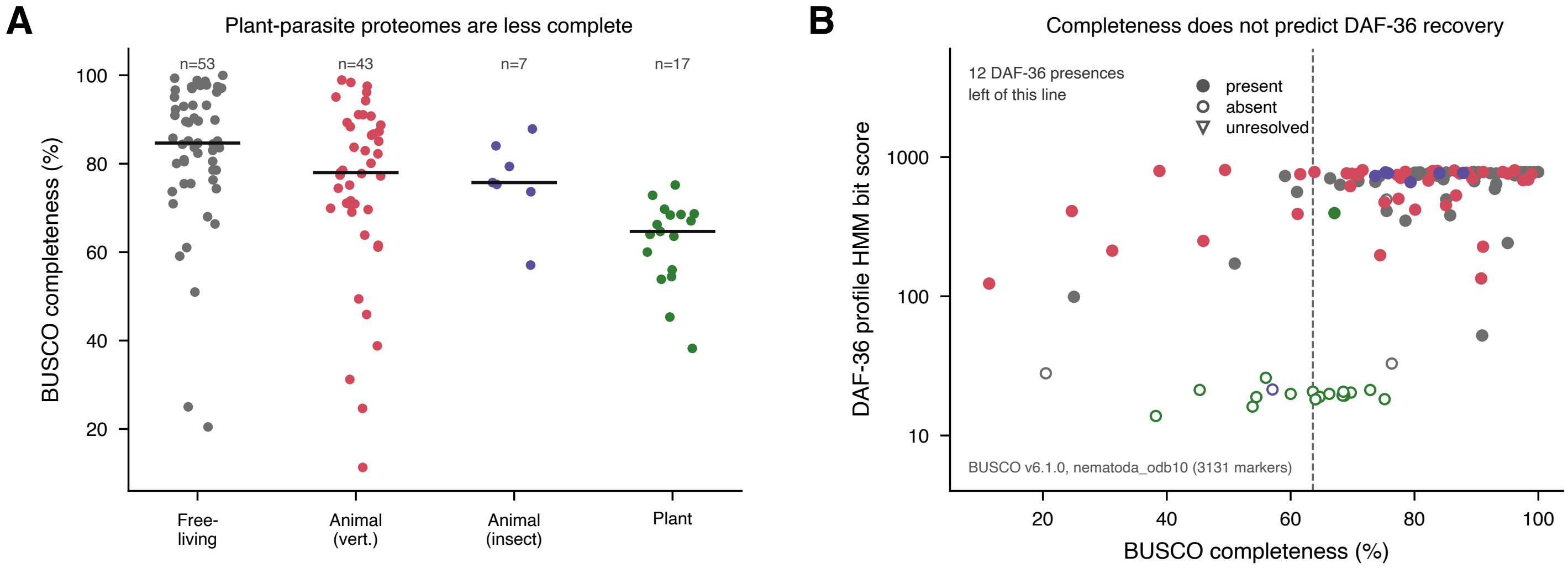

Supplement: Supplementary file 1 [file NIHPP2026.09.03.748874V1-supplement-1.pdf]
