# Supplementary material for: Conservation of Nuclear Receptor DAF-12 with Evidence for Ligand Diversification in Plant-Parasitic Nematodes
Source: bioRxiv. 2026 Sep 7:2026.09.03.748874. Preprint. [Version 1] doi: 10.64898/2026.09.03.748874 (PMC13622478; doi:10.64898/2026.09.03.748874)
Supplement: Supplementary file 2 [file media-1.doc]

(((pos_391-601_PSAMB.scaffold462size50287.g6186.t2_Plectus_sambesii_Clade_C:0.000002,pos_389-487_PSAMB.scaffold6012size10379.g27733.t1_Plectus_sambesii_Clade_C:0.148802)54:0.118267,pos_389-545_PSAMB.scaffold9017size5461.g32038.t2_Plectus_sambesii_Clade_C:0.123350)100:0.264963,(((((pos_736-938_PTRK_0001378700.1_Parastrongyloides_trichosuri_Clade_IV:0.000002,pos_723-932_SRAE_0000032100a.1_Strongyloides_ratti_Clade_IV:0.005058)45:0.005077,pos_734-943_TCONS_00004580.p1_Strongyloides_stercoralis_Clade_IV:0.000002)53:0.001335,(pos_503-712_SPAL_0001591300.1_Strongyloides_papillosus_Clade_IV:0.000001,pos_728-937_SVE_0996600.1_Strongyloides_venezuelensis_Clade_IV:0.000001)99:0.014263)99:0.261849,pos_447-649_RSKR_0001015700.1_Rhabditophanes_sp._KR3021_Clade_IV:0.305499)100:0.423671,(((((((((pos_568-771_nAv.1.0.1.t08097-RA_Acanthocheilonema_viteae_Clade_III:0.000003,(pos_671-877_rna-CJOHNSTONI_LOCUS2016_Cercopithifilaria_johnstoni_Clade_III:0.055329,pos_560-763_nLs.2.1.2.t07694-RA_Litomosoides_sigmodontis_Clade_III:0.033204)82:0.010564)79:0.022751,(pos_674-877_Bm8452.1_Brugia_malayi_Clade_III:0.000001,pos_231-434_BPAG_0001411501-mRNA-1_Brugia_pahangi_Clade_III:0.000003)59:0.000003)50:0.006070,((pos_623-826_EEL_0000446201-mRNA-1_Elaeophora_elaphi_Clade_III:0.027130,(pos_582-785_OVOC2228.1_Onchocerca_volvulus_Clade_III:0.000001,pos_233-436_OOCN_0000985201-mRNA-1_Onchocerca_ochengi_Clade_III:0.000001)100:0.081814)54:0.000002,pos_653-856_mrna-Wban_03033_Wuchereria_bancrofti_Clade_III:0.005162)90:0.024364)62:0.026400,pos_658-861_EN70_12190_Loa_loa_Clade_III:0.037856)66:0.024528,pos_645-855_nDi.2.2.2.t09652_Dirofilaria_immitis_Clade_III:0.015086)98:0.074539,(pos_591-794_sdigi.contig832.g9863.t1_Setaria_digitata_Clade_III:0.039745,pos_281-491_TCLT_0000386601-mRNA-1_Thelazia_callipaeda_Clade_III:0.113933)99:0.062493)94:0.056351,((pos_9-219_ALUE_0001821701-mRNA-1_Ascaris_lumbricoides_Clade_III:0.000001,pos_604-814_AgR054_g014_t02_Ascaris_suum_Clade_III:0.000001)96:0.000002,(pos_614-824_PgB19_g025_t02_Parascaris_univalens_Clade_III:0.005063,(pos_613-823_Tcan_08729.1_Toxocara_canis_Clade_III:0.010194,pos_586-796_ASIM_0001146101-mRNA-1_Anisakis_simplex_Clade_III:0.031102)48:0.005167)60:0.005101)89:0.032099)41:0.004822,pos_504-707_DME_0000943901-mRNA-1_Dracunculus_medinensis_Clade_III:0.155248)96:0.158531,((pos_508-718_EVEC_0001257601-mRNA-1_Enterobius_vermicularis_Clade_III:0.056619,pos_490-700_SMUV_0001002101-mRNA-1_Syphacia_muris_Clade_III:0.101987)100:0.562085,(((((((((pos_659-867_M3Y99_00070200.t1_Aphelenchoides_fujianensis_Clade_IV:0.219479,pos_690-897_M3Y94_00792600.t1_Aphelenchoides_besseyi_Clade_IV:0.186635)100:0.488957,pos_730-937_M3Y97_00254200.t1_Aphelenchoides_bicaudatus_Clade_IV:0.489706)61:0.036194,(pos_474-673_BOKI.060136000.t1_Bursaphelenchus_okinawaensis_Clade_IV:0.077208,(pos_659-858_BXY_1418300.1_Bursaphelenchus_xylophilus_Clade_IV:0.000001,pos_661-860_BXYJ5.060111900.t1_Bursaphelenchus_xylophilus_Clade_IV:0.000001)100:0.120064)100:0.443683)94:0.269653,(pos_853-1048_Bralis.g11736.t1_Bradynema_listronoti_Clade_V:1.377335,pos_514-711_Dd_05346_Ditylenchus_destructor_Clade_IV:0.549534)95:0.389128)32:0.066804,(((pos_747-953_FO519_scaf00256g006040.t1_Halicephalobus_sp._NKZ332_Clade_IV:0.067122,pos_810-1016_MSTRG.10611.1.p1_Halicephalobus_mephisto_Clade_IV:0.060703)100:0.461839,pos_569-770_Pan_g1544.t1_Panagrellus_redivivus_Clade_IV:0.168553)31:0.057367,(pos_624-819_PS1159_v2.g19594.t1_Panagrolaimus_sp._1159_Clade_IV:0.038784,((pos_555-755_PSU_v2.g12710.t1_Panagrolaimus_superbus_Clade_IV:0.028221,pos_548-727_ES5_v2.g15056_Panagrolaimus_sp_ES5_Clade_IV:0.037146)30:0.017728,pos_209-404_PDA_v2.g27050.t1_Panagrolaimus_davidi_Clade_IV:0.015326)38:0.009013)99:0.193263)100:0.672555)31:0.060686,pos_127-328_jg1277_Ditylenchus_dipsaci_Clade_IV:0.837646)46:0.030365,pos_764-986_ACRNAN_scaffold3570.g20078.t1_Acrobeloides_nanus_Clade_IV:0.655886)72:0.090591,(((pos_1097-1292_Hetgly12168.t1_Heterodera_glycines_Clade_IV:0.000001,pos_931-1126_Hsc_gene_16109.t1_Heterodera_schachtii_Clade_IV:0.000001)100:0.207761,(pos_2-186_Gpal_D383_g16826.t1_Globodera_pallida_Clade_IV:0.139380,pos_1407-1605_Gr19_v10_g11452_Globodera_rostochiensis_Clade_IV:0.005177)92:0.078446)100:0.485981,((((pos_676-854_scf7180000423856.g11745_Meloidogyne_floridensis_Clade_IV:0.000001,pos_445-605_scaffold34667_cov245.g21526_Meloidogyne_javanica_Clade_IV:0.006662)100:0.090390,(pos_622-767_scaffold4683_cov250.g8542_Meloidogyne_javanica_Clade_IV:0.022718,(((pos_1134-1315_Minc3s00350g10770_Meloidogyne_incognita_Clade_IV:0.000000,pos_664-845_tig00001238.g15364.t1_Meloidogyne_arenaria_Clade_IV:0.000000)0:0.000000,pos_678-859_tig00001693.g48918.t1_Meloidogyne_arenaria_Clade_IV:0.000000)0:0.000001,pos_641-822_Minc3s01435g23791_Meloidogyne_incognita_Clade_IV:0.000001)94:0.000003)47:0.000002)49:0.000002,((pos_1131-1312_MENT_LOCUS16462_Meloidogyne_enterolobii_Clade_IV:0.000000,pos_1122-1303_MENT_LOCUS40684_Meloidogyne_enterolobii_Clade_IV:0.000000)0:0.000002,pos_1135-1316_MENT_LOCUS24022_Meloidogyne_enterolobii_Clade_IV:0.000001)91:0.005878)91:0.047580,pos_1221-1402_NXFT01000334.1.8827_g_Meloidogyne_graminicola_Clade_IV:0.061590)100:0.341061)100:0.593458)97:0.196226,(((pos_505-710_contig00125.0.25_Steinernema_feltiae_Clade_IV:0.041126,((pos_495-700_L898_g10498.t1_Steinernema_monticolum_Clade_IV:0.031311,pos_1031-1234_L892_g18386.t1_Steinernema_scapterisci_Clade_IV:0.072747)92:0.025162,pos_588-793_Sherm_N.g9231.t3_Steinernema_hermaphroditum_Clade_IV:0.012616)100:0.137440)98:0.161670,(((pos_435-647_Sherm_X.g16888.t3_Steinernema_hermaphroditum_Clade_IV:0.146095,pos_460-672_L893_g20420.t2_Steinernema_glaseri_Clade_IV:0.202339)100:0.567040,pos_483-692_L898_g9012.t1_Steinernema_monticolum_Clade_IV:0.797005)94:0.213223,pos_174-380_L892_g20774.t1_Steinernema_scapterisci_Clade_IV:0.921384)71:0.071806)99:0.182222,((((((pos_542-751_ALDISUDHAUS000001025.mRNA1_Allodiplogaster_sudhausi_Clade_V:0.000001,pos_546-755_ALDISUDHAUS000001054.mRNA1_Allodiplogaster_sudhausi_Clade_V:0.000001)100:0.414692,(pos_475-684_DMAGNUSscaf00050g000997.t2_Diplogasteroides_magnus_Clade_V:0.065332,pos_406-615_KOLU_g446.t1_Koerneria_luziae_Clade_V:0.348446)56:0.056914)33:0.015775,((pos_420-630_LTEXAscaf05760g019681.t3_Levipalatum_texanum_Clade_V:0.188865,((pos_703-912_PRIFISSI000004826.t1_Pristionchus_fissidentatus_Clade_V:0.092130,pos_737-946_PRENTOPHAG000001498.t1_Pristionchus_entomophagus_Clade_V:0.078561)99:0.091341,(((pos_748-957_PREXSPEC000000910.mRNA1_Pristionchus_exspectatus_Clade_V:0.009983,(pos_542-751_PPA28648.1_Pristionchus_pacificus_Clade_V:0.000001,pos_739-948_PARCANUS000000445.t1_Pristionchus_arcanus_Clade_V:0.000001)100:0.000003)100:0.042783,(pos_744-953_PRIMPAX000000819.t1_Pristionchus_maxplancki_Clade_V:0.015400,pos_743-952_TRINITY_c14910_g1_i4_loc1.t1_Pristionchus_japonicus_Clade_V:0.026357)80:0.014154)55:0.016147,pos_505-714_PRIMAYER000000843.t1_Pristionchus_mayeri_Clade_V:0.220478)91:0.045062)77:0.069773)45:0.041764,pos_562-771_MIJAPON000001950.t1_Micoletzkya_japonica_Clade_V:0.074716)39:0.036979)29:0.030323,pos_44-254_PRENTOPHAG000008642.t1_Pristionchus_entomophagus_Clade_V:0.547254)86:0.117336,((pos_515-724_MSPICULIGERA_LOCUS1110.1_Mesorhabditis_spiculigera_Clade_V:0.005172,pos_491-700_MSPICULIGERA_LOCUS12305_Mesorhabditis_spiculigera_Clade_V:0.000002)100:0.504987,pos_464-673_MBELARI_LOCUS7344_Mesorhabditis_belari_Clade_V:0.277895)99:0.213887)65:0.074640,(((((pos_207-416_ACOC_0000064201-mRNA-1_Angiostrongylus_costaricensis_Clade_V:0.005025,pos_446-655_AngVas-contig_57-1.3-mRNA-1_Angiostrongylus_vasorum_Clade_V:0.000003)99:0.021275,((((((pos_477-686_ANCDUO_17434_Ancylostoma_duodenale_Clade_V:0.000000,pos_476-685_Acey_s0032.v2.g6728.t1_Ancylostoma_ceylanicum_Clade_V:0.000000)0:0.000000,pos_460-669_ANCCAN_15412_Ancylostoma_caninum_Clade_V:0.000000)0:0.000001,pos_479-688_Necator_chrX.pre1.g21353.t1_Necator_americanus_Clade_V:0.000001)62:0.000002,(pos_494-703_HPOL_0001414101-mRNA-2_Heligmosomoides_polygyrus_Clade_V:0.014123,pos_476-685_Nippo_chrX.g15301.t1_Nippostrongylus_brasiliensis_Clade_V:0.025529)57:0.011449)43:0.009951,(((pos_47-256_HPLM_0001265201-mRNA-1_Haemonchus_placei_Clade_V:0.000001,pos_728-937_HCON_00164850-00002_Haemonchus_contortus_Clade_V:0.000001)100:0.000002,pos_412-621_TELCIR_05516_Teladorsagia_circumcincta_Clade_V:0.030729)99:0.025200,pos_415-624_DICVIV_07098_Dictyocaulus_viviparus_Clade_V:0.000003)65:0.004977)38:0.010188,(pos_452-661_CYNAS_LOCUS21369_Cylicocyclus_nassatus_Clade_V:0.019301,pos_201-389_SVUK_0001133001-mRNA-1_Strongylus_vulgaris_Clade_V:0.046246)100:0.044954)20:0.000003)40:0.010302,pos_77-286_OESDEN_04175_Oesophagostomum_dentatum_Clade_V:0.000002)99:0.115766,(pos_591-797_JU1783_LOCUS10797.2_Auanema_sp._JU1783_Clade_V:0.382413,(((pos_639-850_DCO_004589_Diploscapter_coronatus_Clade_V:0.008685,pos_639-850_WR25_16540.1_Diploscapter_pachys_Clade_V:0.009088)24:0.006204,pos_639-850_DCO_004590_Diploscapter_coronatus_Clade_V:0.001415)100:1.723311,(pos_480-689_OTIPU.nOt.2.0.1.t01766_Oscheius_tipulae_Clade_V:0.000001,pos_658-867_Oti_16230_t_Oscheius_tipulae_Clade_V:0.000001)100:0.161524)60:0.141477)75:0.117162)87:0.136821,(pos_120-303_Cang_2012_03_13_00091.g4056.t2_Caenorhabditis_angaria_Clade_V:0.930486,(pos_484-693_rna-CAUJ_LOCUS4595_Caenorhabditis_auriculariae_Clade_V:0.345021,((pos_487-698_CSP38.g8805.t1_Caenorhabditis_quiockensis_Clade_V:0.054076,(pos_558-769_CSP21.g10182.t2_Caenorhabditis_parvicauda_Clade_V:0.048896,pos_473-684_CBOVI.g3971.t1_Caenorhabditis_bovis_Clade_V:0.200931)81:0.036298)43:0.015068,(pos_468-679_Cang_2012_03_13_00091.g4057.t1_Caenorhabditis_angaria_Clade_V:0.056734,(pos_494-705_CSP31.g25010.t3_Caenorhabditis_uteleia_Clade_V:0.097866,(pos_1-128_CBN20181.1_Caenorhabditis_brenneri_Clade_V:0.238746,(((pos_475-686_CSP32.g2065.t1_Caenorhabditis_sulstoni_Clade_V:0.089553,pos_495-706_CJA11532.1_Caenorhabditis_japonica_Clade_V:0.009789)87:0.017228,((pos_499-710_CSP39.g8512.t1_Caenorhabditis_waitukubuli_Clade_V:0.012166,pos_471-682_CSP29.g15982.t1_Caenorhabditis_becei_Clade_V:0.017168)73:0.012569,pos_468-679_CSP28.g5501.t1_Caenorhabditis_panamensis_Clade_V:0.020129)79:0.019919)66:0.012877,(((((pos_509-720_FL83_01282.3_Caenorhabditis_latens_Clade_V:0.000000,pos_775-986_CRE07237.1_Caenorhabditis_remanei_Clade_V:0.000000)0:0.000001,pos_466-677_GCK72_023616.t1_Caenorhabditis_remanei_Clade_V:0.000001)100:0.005801,(((pos_503-712_Csp5_scaffold_00063.g3034.t1_Caenorhabditis_sinica_Clade_V:0.025561,pos_706-917_CSP40.g20318.t2_Caenorhabditis_tribulationis_Clade_V:0.020076)66:0.004600,pos_507-718_CSP26.g3116.t1_Caenorhabditis_zanzibari_Clade_V:0.000003)74:0.007994,(pos_565-776_CBG04512a.1_Caenorhabditis_briggsae_Clade_V:0.000003,pos_492-703_Cni-daf-12.1_Caenorhabditis_nigoni_Clade_V:0.000002)100:0.013785)78:0.012424)52:0.009775,(pos_527-738_CBN31767.1_Caenorhabditis_brenneri_Clade_V:0.035327,pos_769-980_Csp11.Scaffold629.g13785.t2_Caenorhabditis_tropicalis_Clade_V:0.017670)63:0.002758)53:0.005362,(pos_526-737_Sp34_X0074510.t1_Caenorhabditis_inopinata_Clade_V:0.034575,(cele_daf12_LBD:0.000001,pos_539-750_F11A1.3a.1_Caenorhabditis_elegans_Clade_V:0.000002)100:0.025633)73:0.009799)23:0.000002)63:0.008622)95:0.043852)85:0.036584)36:0.009979)63:0.053313)75:0.080016)100:0.411243)87:0.085005)100:0.168303)80:0.122089)70:0.050605)68:0.107976)64:0.065098)100:0.264963);
